# Network Stability Is a Balancing Act of Personality, Power, and Conflict Dynamics in Rhesus Macaque Societies

**DOI:** 10.1371/journal.pone.0022350

**Published:** 2011-08-03

**Authors:** Brenda McCowan, Brianne A. Beisner, John P. Capitanio, Megan E. Jackson, Ashley N. Cameron, Shannon Seil, Edward R. Atwill, Hsieh Fushing

**Affiliations:** 1 California National Primate Research Center, University of California Davis, Davis, California, United States of America; 2 Department of Population Health and Reproduction, School of Veterinary Medicine, University of California Davis, Davis, California, United States of America; 3 Department of Anthropology, Pennsylvania State University, University Park, Pennsylvania, United States of America; 4 Department of Psychology, University of California Davis, Davis, California, United States of America; 5 Department of Statistics, University of California Davis, Davis, California, United States of America; Université Paris 13, France

## Abstract

Stability in biological systems requires evolved mechanisms that promote robustness. Cohesive primate social groups represent one example of a stable biological system, which persist in spite of frequent conflict. Multiple sources of stability likely exist for any biological system and such robustness, or lack thereof, should be reflected and thus detectable in the group's network structure, and likely at multiple levels. Here we show how network structure and group stability are linked to the fundamental characteristics of the individual agents in groups and to the environmental and social contexts in which these individuals interact. Both internal factors (e.g., personality, sex) and external factors (e.g., rank dynamics, sex ratio) were considered from the level of the individual to that of the group to examine the effects of network structure on group stability in a nonhuman primate species. The results yielded three main findings. First, successful third-party intervention behavior is a mechanism of group stability in rhesus macaques in that successful interventions resulted in less wounding in social groups. Second, personality is the primary factor that determines which individuals perform the role of key intervener, via its effect on social power and dominance discrepancy. Finally, individuals with high social power are not only key interveners but also key players in grooming networks and receive reconciliations from a higher diversity of individuals. The results from this study provide sound evidence that individual and group characteristics such as personality and sex ratio influence network structures such as patterns of reconciliation, grooming and conflict intervention that are indicators of network robustness and consequent health and well-being in rhesus macaque societies. Utilizing this network approach has provided greater insight into how behavioral and social processes influence social stability in nonhuman primate groups.

## Introduction

Stability in biological systems requires the evolution of mechanisms to promote or maintain this stability in spite of the inevitable conflict among group members [1]. Social groupings among animals are one example of a stable biological system. Group-living animals gain benefits from doing so, such as protection from predators, access to coalitionary partners, and improved access to food resources [2], . However, competition among group members is inevitable, because conspecifics seek out similar resources (i.e., mates, food, alliance partners). Such intrinsic conflict among group members could lead to social instability. Therefore, the persistence of stable social groups in primate societies indicates that robustness mechanisms must have evolved for mitigating these costs and thereby counteracting this inherent instability.

### Mechanisms of group stability

Multiple sources of stability likely exist for any biological system. Sources of social stability in animal social groups include conflict resolution or reconciliation [5], conflict intervention by third parties [6], group size and composition [7], [8], and kinship structure [9]. Precisely which factors play a role in group stability may be dependent upon the social system of a given species.

Conflict intervention by third parties which results in termination of the conflict is a particularly intriguing source of stability both because of its obvious utility in reducing the frequency and severity of aggression and its inherent risk to the intervener. Flack and colleagues identified impartial conflict intervention behavior, in which individuals intervene on others' conflicts without favoritism to either opponent [10], as a mechanism of group stability in a captive group of pigtailed macaques (*Macaca nemestrina*), showing that temporary removal of key interveners increases group-level rates of aggression [6] and results in reorganization of social niches (i.e., individuals form smaller and less diverse networks; lower degree of integration within the group network) [11]. In order for conflict interference behavior to evolve as a mechanism of group stability, the potential costs of intervention must be sufficiently low for interveners. In captive pigtailed macaques, the most frequent and successful interveners were four adults with high social power [6], a measure of the extent to which group members agree over the status of dominant individuals and their perceived capability to use force [12]. Thus, individuals with high social power have a low risk of retaliation when intervening and therefore a low cost of intervention.

Although conflict intervention appears to be a mechanism of group stability in pigtailed macaques, its applicability to other species has not been evaluated. Since conflict intervention behavior appears to be performed primarily by a small subset of very powerful individuals [13], the utility of intervention as a mechanism of stability may require a highly skewed group power structure, which may not be present in other animal societies. Furthermore, the factors that influence which individuals attain high social power and whether they become key interveners remain unknown.

### Who becomes a key intervener?

Flack and colleagues report that four individuals with high social power performed the majority of successful interventions, indicating that social power is a requirement to be a key intervener. These individuals were also high-ranking, and most (3 of the four) were males, suggesting that only high-ranking males tend to be successful interveners. Macaque males are larger than females [14], thus males' physical size and strength may give them a greater ability to successfully intervene upon the conflicts of others [10]. Indeed, our research group has already shown that high-ranking males in our rhesus groups are the most successful interveners [15]. However, high-ranking males are not the only successful interveners, suggesting that factors other than rank and sex may affect which individuals become key interveners as well which individuals have high social power.

### Personality, social power, and intervention behavior

Although third-party intervention behavior may be important to the maintenance of group stability, it remains unknown which individuals become key interveners for the group because two important questions remain unanswered: (1) which individuals attain high social power and (2) which of those with high social power become key interveners. We hypothesize that the answer to these questions may lie in differences in personality.

Personality likely contributes significantly to individuals' perceived ability to successfully use force (i.e., social power) and likelihood of getting involved in the affairs of others (i.e., intervention behavior). The vast majority of personality research in nonhuman primates has been conducted on rhesus macaques (approximately 40% [16]) and four personality dimensions have typically been identified: Sociability, Boldness, Excitability, and Equability [16]. In anthropomorphic terms, individuals with high social power may be feared, respected, or well-liked by the rest of the group because each of these could result in a group consensus or individuals' agreement that the individual is powerful. Highly aggressive individuals might be regarded as being powerful, but so too might equable individuals. Personality dimensions such as aggressiveness, sociability, equability, and predictability can all influence whether an individual is likely to intervene and whether s/he is respected by other group members. For example, among adult rhesus macaques, personality traits such as Sociable, Confident, and Equable have been found to consistently correlate with the tendency to interact affiliatively with others, the tendency to be aggressive toward others, and the tendency to interact passively, respectively [17]. Furthermore, males high in Excitability are inconsistent in their social behavior. We predict that individuals with different personalities may acquire high social power, but for different reasons, and that these differing methods of power acquisition will result in variation in intervention behavior.

The purpose of this study therefore was to examine the effects of personality, social power and conflict intervention on social group stability (as measured by rates of wounding and social relocation) at the individual and population levels in rhesus macaque societies using a network approach. The network approach was selected to focus the unit of analysis on patterns in relationships rather than rates of behaviors given the importance of relationships to social stability in macaques [6], [8], [9], [18]. Indeed social robustness mechanisms should be reflected in network structure at multiple levels of analysis. The focus on patterns of relationships is what distinguishes social network analysis from other analytical techniques [19]. Social network analysis thereby provides insight into the patterns of social relationships through quantitative measures such as “degree”, “reciprocity”, “betweenness”, and “fragmentation” and was used along with statistical modeling in this study to test whether perturbations in these patterns of relationships (due to external or internal sources) have significant positive or negative consequences on social structure and stability.

## Methods

### Data collection

#### Behavioral and attribute data collection

Seven social groups comprised of 69 matrilines and 1152 individuals, uniquely dyemarked and tatooed for identification, were the focus of this study at the California National Primate Research Center. These social groups (one per half-acre cage ranging from 108–197 individuals) were studied between June 2008 and December 2009 for a total of ∼1400 hours (See Table 1 for observation hours by group). Data were collected using event or scan sampling [20] for six hours on four days per week for one week of each month during each group's study period. Data were collected on the affiliative (e.g., groom, reconcile, huddle, rump present), submissive (e.g., move away, run away, scream, silent bared teeth display, rump present) and aggressive interactions (e.g., threat, chase, bite) among individuals within each group comprising a total of 112,189 event samples (conflict, status signaling, reconciliation) and 24,621 scan samples (grooming and huddling) in the data set (inter-observer reliabilities had mean of 91% agreement and a standard deviation of 3%; range: 86–94%; kappa  = 0.65, *p*<0.0001 across three observers) [8], [9]. Data were also collected on group and family attributes including group size, proportion of different sex/age classes, number of matrilines, sex ratio of adult female to unrelated adult male (to alpha and beta matrilines), average size of matrilines, mean of kin coefficients representing the average degree of relatedness among individuals within matrilines, as well as the number of wounds (# wounds requiring hospitalization in group) and social relocations (# animals permanently removed from group due to either extreme aggressiveness or as repeated targets of deleterious aggression assessed by trained observers) as measures of group stability across each study period (see Table 1 for a subset of these measures by group).

**Table 1:** Attributes of rhesus groups observed in study.

Group	Average Group Size	Obs. Hours	Sex Ratio (F/M)	ADR*	W/SR**
1	177.6	182.05	2.59	0.29	45/6
5	136.6	251.80	6.43	0.16	175/26
8	156.9	231.80	4.92	0.16	78/6
10	164.9	178.10	20.89	0.12	289/43
14	108.3	226.33	13.97	0.17	19/1
16	149.4	163.92	41.19	0.10	223/72
18	197.2	175.53	8.43	0.18	63/1

*Average degree of relatedness.

**Wounds/Social Relocation.

#### Personality assessment

Personality was evaluated for each individual in the alpha matriline as well as the alpha and beta males to assess its effect on group stability in 6 of the 7 groups (N = 60; one group had to be disbanded before assessment could be conducted). Two experienced observers rated all subjects (N = 60) according to 29 descriptors (e.g., bold, lazy, cautious, affiliative) adapted from Capitanio [17] and Stevenson-Hinde [21]. While observers strictly avoided discussion of *individual subjects,* observers did discuss their subjective interpretations of *each trait* in order to develop similar conceptualizations, particularly of how each trait might manifest in an animal's behavior.

Observers supplemented their personal experience and knowledge of the study subjects' behavioral tendencies, where applicable, with two 10-minute focal animal samples, one in the morning (8–11AM) and one in the afternoon (1–5PM). Both of each subject's focal sessions were completed within one week, but not on the same day. During focals, the subject's affiliative and aggressive interactions with adult group members were recorded on hand-held computers using methods described above and in Beisner et al. [8], [9]. Each observer individually rated all subjects on the 29 descriptors using a seven-point scale (1 =  total absence of trait, 7 =  extreme manifestation of trait). A score of 0 was given when the observer did not have enough information to score a particular trait.

Data for personality were collected after all other data collection was completed for the study. Some groups had not been observed for several months (N = 3) and others approximately two months prior to the assessment (N = 3). One observer was familiar with all of the groups and the other only with three of the six groups. Therefore, we are confident that personality assessment was conducted independently of the study data.

### Data analysis

#### Social network analyses

Please see Supporting Information S1 for a detailed description of the social network measures used and outlined in Table 2.

**Table 2:** List of measures used in study.

Individual Rank	Social rank of each individual within cage
Dominance Discrepancy	Degree of separation in dominance of one individual to others in group
Social Power	Weighted first-order entropic-like measure representing number of signals of subordination received/number of signals of submission received by an individual
Intervention Success	Proportion of interventions that successfully ended conflict x # successful interventions by individual
Intervention Out-degree	Diversity of individuals on which an individual initiated interventions whether successful or not
Groom Betweenness Centrality	Degree to which each individual links others in a grooming network
Reconciliation In-degree	Diversity of individuals from which an individual receives reconciliation through grooming
Hierarchy Discrepancy	Measure of group hierarchy structure using – natural log fit of the # of submission signals against dominance rank representing degree of variance in number of submissions received across individuals in a group
Average Power	Average of social power across individuals for each group
Average Intervention Success	Average of intervention success across individuals for each group
Groom Reciprocity	Measure of degree of reciprocity in grooming network for each group
Reconciliation Clustering Coefficient	Measure of connectedness of reconciliation network for each group
Average Conflict Length	Average number of transactions of conflict in an event
Average Personality	Average scores of personality types across individuals for each group
Average Contact Aggression	Average of aggression involving contact (e.g., hit, bite, etc).

#### Personality analysis

The personality analysis method described in Capitanio [17] was followed with a few modifications. Scores generated from the observers were subjected to two types of filtering. First, personality scores that showed no variation across subjects were dropped. Next, a comparison of the two observers' scores for the remaining personality items were compared and those differing by more than two points for more than 25% of the individuals were dropped. Finally an average of the two observers for the remaining scores was obtained and these values were subjected to factor analysis using a promax rotation in S-Plus 6.0 [22].

This analysis resulted in four factors (positive loadings of individual traits: Factor 1: Bold, Confident, Direct; Factor 2: Unpredictable, Impulsive, Reckless, Aggressive, Excitable, Active, Vigilant; Factor 3: Tolerant, Calm, Gentle, Understanding, Popular; Factor 4: Affiliative, Warm; Cronbach's alpha  = 0.92), which, as in previous rhesus studies, correspond to four personality types [17]: Bold, Excitable, Equable and Warm. The scores, ranging from −3.0 to 3.0 for each factor were used as variables in all subsequent analyses.

#### Statistical analyses

At the individual level, network measures were used to statistically examine, using multi-level mixed effects Poisson or Gaussian regression in Stata 11 [23], the association between individual attributes such as sex, rank, dominance discrepancy, and personality type, social network structures and patterns of cooperation (grooming, reconciliation) and conflict (contact aggression received) behavior within groups. A nested random effect of matriline within cage was included to account for any dependencies among data points (individuals) due to these variables. For individual level analyses, individuals were included in the analysis if they were adults and if they received at least one signal of subordination during the course of the study. We chose to filter on this basis to avoid the skew toward zero that would be generated by including the large number of individuals for which the rate of receiving signals of subordination was absent (or zero). This filtering therefore excluded individuals that were either relatively asocial or those from lower ranking matrilines. For group level analyses, all adult individuals were included with two exceptions. Group level analyses of averaged intervention success and averaged social power included only those individuals as described above. We note however that analyses were repeated with all individuals (N = 1131) and showed almost identical results to that of the subset both at the individual and group levels.

At the group level, network measures were used to statistically examine, using Poisson or Gaussian regression analysis in Stata 11, the associations among the individual and family attributes, social network structures, rates of wounding and social relocations across the seven enclosures. Proportion variables (e.g., displacement fragmentation, reconciliation clustering coefficient, groom reciprocity) were Arcsin transformed prior to analysis when they were used as outcomes [24]. Due to sample size/power issues for the group level (N = 7), we were limited to small models but average group size (averaged across daily measure of period of observation) was included as a fixed effect in each analysis to account for the potential effect of group density in our analyses and observation time (hours observed for each group) as an exposure variable to statistically account for the differences in observation times across groups (see Table 1).

In all analyses, a term's inclusion in the model was set at α = 0.05 unless otherwise noted, and the best fit model was chosen using the AIC approach. Models having a difference in AIC less than or equal to two were considered equivalent [25]. For the individual level models, measures of goodness-of-fit were also evaluated by examining observed versus predicted values.

## Results

### Individual level

#### Intervention success

**Figure 1:**
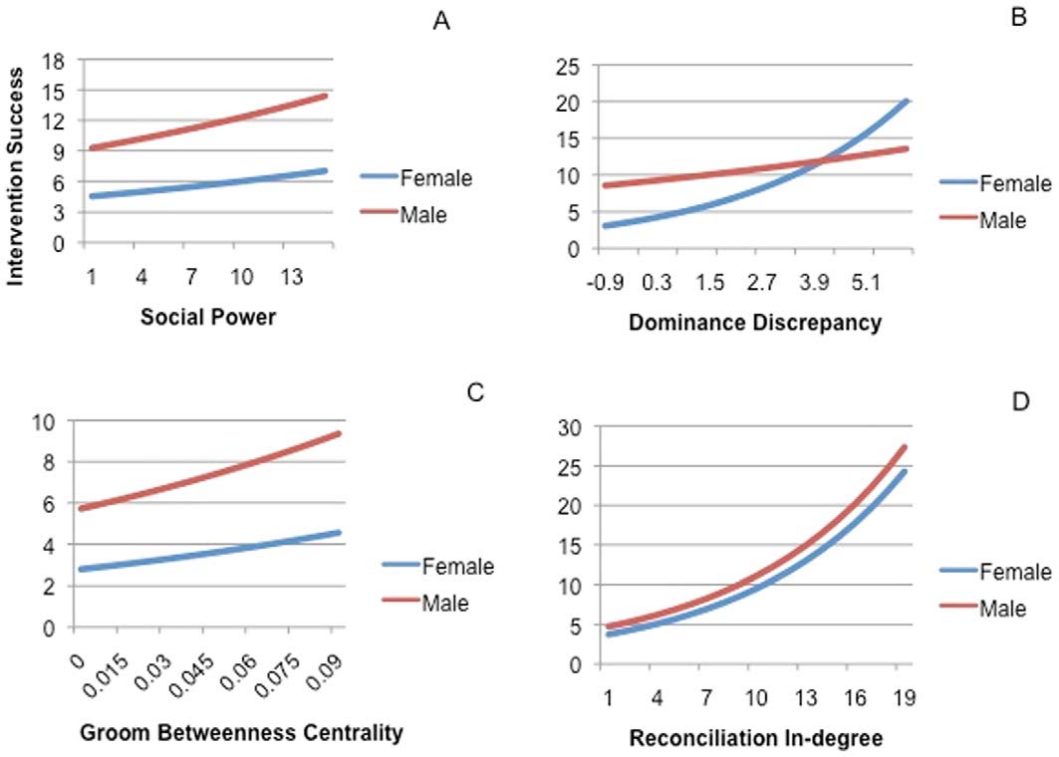
Relationship between intervention success and (a) social power, (b) dominance discrepancy, (c) groom betweenness centrality and (d) reconciliation in-degree for males and females at the individual level.

Table 3 presents a summary of results found at the individual level. The best fit AIC model (Wald  = 425.9, p<0.0001, R^2^ = 0.66, N = 322) with the outcome of intervention success at the individual level included social power, rank and dominance discrepancy, groom betweenness centrality and reconciliation in-degree with a sex by dominance discrepancy interaction. Greater intervention success was evident for individuals with higher social power (ß = 0.03, p = 0.002), greater dominance discrepancy (ß = 0.26, p<0.0001), greater bridging of other individuals though grooming (groom betweenness centrality; ß = 8.16, p = 0.023) and receipt of reconciliation from a higher diversity of individuals (reconciliation in-degree; ß = 0.05, p<0.0001) (see Figure 1). Rank (1 being highest ranking) showed a negative association with intervention success (ß = −0.02, p<0.0001). Males exhibited a significantly higher intervention success than females (ß = 0.33, p = 0.001) and females required higher dominance discrepancy than males to achieve similar levels of intervention success (ß = −0.17, p<0.0001). No significant interactions were found for social power, reconciliation in-degree or groom betweenness centrality and sex.

**Table 3:** Individual-level results.

	Intervention Success			Social Power		
Data	AllN = 322	MalesN = 92	α- matrilineN = 53	AllN = 322	MalesN = 92	α- matrilineN = 53
Social Power	+	NA^b^	+ (interaction)	NA	NA	NA
Rank^a^	-	-	NA	-	-	NA
Dominance Discrepancy	+	NA	NA	+	NA	NA
Groom Betweenness	+	NA	NA	+	NA	NA
Reconciliation In-degree	+	NA	NA	+	NA	NA
Sex (male)	+	NA	+ (interaction)	+	NA	+ (interaction)
Unrelated to α, β matrilines	NA	+	NA	NA	ns	NA
Bold personality	NA	NA	+ (interaction)	NA	NA	+
Equable personality	NA	NA	+ (interaction)	NA	NA	ns (interaction)
Excitable personality	NA	NA	ns (interaction)	NA	NA	+

Plus (+) signs indicate significant main effects with a positive relationship to the dependent variable. Minus (-) signs indicate significant main effects with a negative relationship to the dependent variable. The letters ‘ns’ indicate non-significant main effects. The word ‘interaction in parentheses indicates a predictor involved in significant interaction terms, regardless of the significance of the main effect. The data set for ‘α- matriline’ is subset of data that only included individuals from the highest-ranking matriline (alpha) and the alpha and beta males.

a 1 being highest ranking.

b NA means no association.

#### Social power

As with intervention success, social power was also associated with patterns of affiliation and dominance (Wald = 4∼6∼9, p<0.0001, R^2^ = 0.89, N = 322). In the best fit AIC model, individuals with high social power were higher in rank (ß = −0.02, p<0.0001), had greater dominance discrepancy (ß = 0.39, p<0.0001), exhibited greater bridging of other individuals though grooming (groom betweenness centrality; ß = 8.40, p = 0.005), and receive reconciliations from a higher diversity of individuals (reconciliation in-degree: ß = 0.03, p = 0.025). Males also exhibited higher social power than females (ß = 0.39, p = 0.002), but sex showed no significant interactions with the other variables in the model.

#### Effect of male relationship to high-ranking matrilines on social power and intervention success

Using a subset of the data that only included males (N = 92), we determined whether relatedness of adult males to the females in the groups had an effect on their social power or their ability to intervene successfully in others' conflicts. Males unrelated to high-ranking [alpha or beta] matrilines were 55% more likely to intervene successfully (ß = 0.44, p = 0.024) than related males. Unrelated males did not show significantly higher social power than related males (ß = −0.32, p = 0.45).

#### Effect of personality of high-ranking individuals on social power and intervention success

**Figure 2:**
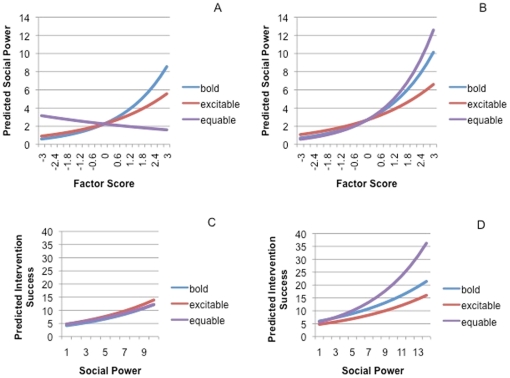
Relationship between (a) social power and personality types in females from alpha matriline, (b) social power and personality types in alpha and beta males, (c) social power and intervention success by personality type in females from the alpha matriline, and (d) social power and intervention success by personality type in alpha and beta males.

Using a subset of data that only included individuals from the highest-ranking matriline (alpha) and the alpha and beta males assessed for personality (N = 53), we examined the importance of personality on social power and intervention success. In the best fit AIC model for social power, three of the four personality types (bold, excitable, equable) showed a positive relationship with social power (Wald  = 172.3, p<0.0001, R^2^ = 0.87; boldness: ß = 0.44 p<0.0001; excitability: ß = 0.30, p = 0.001; equability: ß = −0.11, p = 0.413, sex by equability interaction: ß = 0.63, p = 0.009) in alpha/beta males but only two personality types (bold, excitable) showed a positive relationship with social power in females from the alpha matriline (see Figure 2). These females were also less likely to have equable personality in comparison to the alpha/beta males (ß = −1.19, p<0.0001).

For intervention success (Wald  = 94.4, p<0.0001, R^2^ = 0.83), bold (ß = 0.53, p<0.0001) and equable (ß = 0.21, p = 0.003), but not excitable (ß = −0.019, p = 0.75), individuals had higher intervention success. When social power and sex were included (best fit AIC model: Wald  = 300, p<0.0001, R^2^ = 0.87), the social power × personality x sex interaction indicated that equable high-ranking males were more likely to intervene successfully when they had high social power (ß = 0.07, p = 0.425; power x equable interaction: ß = 0.03, p = 0.031; sex x equable interaction: ß = 0.34, p = 0.035; see Figure 2). Bolder males and females with high social power intervened more successfully than bold males with less social power (ß = 0.52, p<0.0001; power x bold interaction: ß = −0.02, p = 0.253), as did excitable individuals (ß = 0.07, p = 0.425; power x excitable interaction: ß = −0.06, p = 0.001), but *much less so* than equable high-ranking males (see Figure 2). Equable high-ranking females showed no difference in intervention success than bold or excitable individuals. In addition, bold (ß = 0.39, p<0.0001) and equable (ß = 0.22, p = 0.001) males and females showed a positive association with intervention degree (whether successful or not) whereas excitable individuals did not (ß = 0.09, p = 0.175).

### Group level

**Figure 3:**
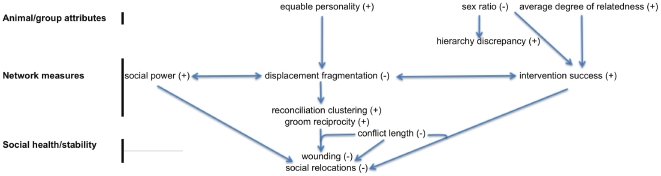
Schematic of the relationships found among animal/group attributes, network measures and social stability as measured by rates of wounding and social relocation. A “+” means that a positive value had the subsequent effect and a “-“ means that a negative value has the subsequent effect. For example, a positive (higher) value of intervention success had negative effect (lowered rates) on wounding and social relocations. Conflict length was associated with multiple paths of social stability (see text).

Group level network measures reflected the patterns found at the individual level and were associated with group stability as measured by rates of wounding requiring hospitalization and social relocation of individuals. Figure 3 provides a schematic of the relationships found among sex ratio (adult females to unrelated adult males to alpha/beta matriline), hierarchy discrepancy, personality, social network measures and rates of wounding and social relocations at the group level.

#### Effects of group attributes on network measures and group stability

Groups with a higher ratio of females per unrelated male exhibited higher rates of both traumas (ß = 0.04, p<0.0001) and social relocations (ß = 0.06, p<0.0001) and exhibited less hierarchy discrepancy (ß = −0.85, p<0.0001), lower intervention success (ß = −0.07, p = 0.058), and lower average equable personality (ß = −0.04, p = 0.006). Average degree of relatedness exhibited a negative association with rates of trauma (ß = −15.86, p<0.0001) and social relocation (ß = −27.52, p<0.0001) as well as a positive association with intervention success (ß = 17.54, p = 0.025). Average equable personality (ß = −0.42, p = 0.009) and hierarchy discrepancy (ß = −0.02, p = 0.002) showed a negative association with displacement fragmentation, a measure of the connectedness or redundancy of dominance interactions within groups.

#### Relationships among network measures and group stability

Average intervention success (ß = −0.14, p = 0.015) and average social power (ß = −0.41, p = 0.001) each showed a negative association with displacement fragmentation. Greater displacement fragmentation was further associated with higher rates of trauma (ß = 3.45, p<0.0001) and social relocation (ß = 6.14, p<0.0001). Lower rates of trauma and social relocation were also associated with more reciprocal grooming (groom reciprocity; ß = −12.47, p<0.0001; ß = −2.76, p<0.0001) and greater connectedness in reconciliation behavior (reconciliation clustering; ß = −12.47, p<0.0001; ß = −22.08, p<0.0001). Average social power was negatively associated with trauma (ß = −0.16 p = 0.006) and social relocations (ß = −.69 p<0.0001), as was higher average intervention success (ß = −0.37, p<0.0001; ß = −0.61, p<0.0001). Average conflict length was negatively associated with groom reciprocity (ß = −2.70, p = 0.003), reconciliation clustering ß = −3.08, p = 0.017) and intervention success (ß = −0.07, p = 0.038) and positively associated with displacement fragmentation (ß = 0.84 p<0.0001), rates of trauma (ß = 1.06, p<0.0001) and social relocation (ß = 3.96, p<0.0001). However, rates of trauma and social relocation were *not associated* with contact aggression rate at the group level (ß = −45.68, p = 0.568; ß = −1.46, p = 0.945).

## Discussion

These results yielded three main findings that are supported by the combination of the individual and group-level results. First, successful third-party intervention behavior is a mechanism of group stability in rhesus macaques as in Flack's pigtailed macaques [11]. Second, personality is the primary factor that determines which individuals perform the role of key intervener, via its effect on social power and dominance discrepancy. Finally, individuals with high social power are not only key interveners but also key players in grooming and reconciliation networks.

### Third-party intervention is a mechanism of group stability

Among seven captive rhesus groups, successful intervention by key individuals was associated with greater group stability. First, individuals with high social power had higher dominance discrepancy and higher intervention success. Individuals with high discrepancy in dominance and social power are less likely to be challenged during a conflict so are more likely to be successful in stopping a conflict. This is true at the group-level, where groups with greater hierarchy discrepancy have more intervention success because individuals contributing to this greater discrepancy in these groups are less likely to be challenged. Furthermore, groups with higher average intervention success, more redundancy in dominance interactions (lower displacement fragmentation) and higher average social power had less wounding and social relocation. These results are in agreement with our previous findings [18] and those of Flack and colleagues [11], [13] and extend our knowledge of this robustness mechanism by demonstrating that how much more dominant an individual is over others (dominance discrepancy) influences the likelihood of successful intervention. Although rank and social power are not equivalent measures, it seems that high social power may, in part, descend from a high degree of dominance discrepancy.

### Personality as a primary determinant of key interveners

Despite the positive relationship between social power and intervention success, high social power does not always lead to intervention success because personality influences this relationship. Three of the four personality types in high-ranking males had a positive association with social power, suggesting that individuals of different personality receive signals of subordination for different reasons. Bold individuals likely receive signals because they are approaching individuals with greater frequency. Excitable individuals receive signals because they are unpredictable; other group members may give signals of subordination by default to avoid unpredictable aggression. Equable individuals likely receive signals because they are respected and popular members of the group. Interestingly, only two of the four personality types, bold and excitable, showed a positive relationship with social power in females, suggesting that high-ranking females are less likely to exhibit equable personalities than high-ranking males.

Personality and intervention success were also positively associated but differed for high-ranking males and females. Equable males with high social power were much more successful interveners than either bold or excitable males. Social power showed almost no relationship with intervention success for excitable males and a weaker relationship for bold individuals than equable males. Females showed no differences in the relationship between intervention success and social power across the bold, excitable and equable personality types, and exhibited much lower rates of intervention success than males. High-ranking bold and equable individuals regardless of sex were the individuals that intervened in others' conflicts. Excitable individuals did not intervene. These results suggest that high-ranking equable males with high social power are the most successful interveners, which serve to minimize conflict duration and potential wounding.

### Sex and relatedness influence intervention behavior

Males were better interveners than females and needed less dominance discrepancy to achieve similar levels of success than females, which may be attributed to several factors. First, males' greater success may be due to difference in size and strength as a result of sexual dimorphism in rhesus. Second, females' ranks are established and maintained through alliances with kin and nonkin, whereas males' ranks are more often based upon age, body size, or group tenure [26], Therefore, any conflict involving females is likely to have an impact, either directly or indirectly, on females' individual or matriline rank and is less likely to influence males' rank. As a result, females may often intervene to exacerbate a conflict, whereas male interventions should less often result in the exacerbation of a conflict. Finally, since females belong to matrilines, their kin might modify the social power of females. Indeed, the only female policer in Flack and colleagues' study [13] was the alpha female, whose matriline included no other adult females (J. Flack, personal communication), suggesting that presence of kin may decrease females' social power. Males' relatedness to females further influences their intervention success. Males unrelated to the alpha and beta matrilines were more successful at intervening than related males but required more dominance discrepancy to achieve higher success rates. The greater success of unrelated versus related males might be attributed to related males using a different strategy (i.e., kin alliances as opposed to age, body size, competitive ability, or group tenure) for achieving high rank and influential positions within the male network than unrelated males [8]. At the group-level, greater sex ratio of unrelated males to females, higher averaged degree of relatedness, and higher number of high-ranking equable individuals lead to greater social stability in rhesus groups.

### Overlap in network roles: key interveners are also key grooming and reconciliation partners

Individuals with higher social power and intervention success were prominent in the grooming network by connecting other individuals in the grooming network (groom betweenness centrality). They also received more reconciliation from a diversity of individuals (reconciliation in-degree) than those with lower social power and intervention success. Grooming serves many social functions, such as relieving tension [27] and establishing/maintaining important relationships [28]. High social power likely makes an individual an attractive grooming partner, perhaps to gain/repair a valuable alliance relationship or because proximity to a high powered individual offers one protection from being a target of aggression. High rates of reconciliation with successful interveners that are also high in social power suggests that maintaining a good relationship with key interveners is beneficial to group members.

### Relationship between individual-level and group-level

For nearly all measures, individual-level patterns of relationships are reflected in group-level patterns and point to the robustness mechanisms underlying the group-level patterns as well as social stability in rhesus groups. However, group-level patterns are self-organizing and not simply the sum of individual behaviors. Social power did not exhibit an association with intervention success at the group level as it did at the individual level. This lack of relationship is likely because individuals are receiving signals of subordination for a variety of reasons, which depends in part on the personality of those receiving these signals. Furthermore, the average amount of contact aggression did not predict wounding or social relocations and points to the idea that *patterns in relationships* among individuals may be more indicative of group stability than simply *rates of behavior* summed across individuals.

### Conclusion

The results from this study provide sound evidence that individual and group characteristics such as personality, social status and sex ratio interact to influence network structures such as patterns of reconciliation, grooming and conflict intervention that are indicators of network robustness and consequent health and well-being in rhesus macaque societies. They also illustrate that rhesus societies, like human societies, are self-organizing entities that do not just equal the sum of their parts. Utilizing this network approach has provided greater insight into how behavioral and social processes influence social stability in nonhuman primate groups.

## Supporting Information

Supporting Information S1 (DOCX)
