# Supplementary material for: Network Stability Is a Balancing Act of Personality, Power, and Conflict Dynamics in Rhesus Macaque Societies
Source: PLoS One. 2011 Aug 3;6(8):e22350. doi: 10.1371/journal.pone.0022350 (PMC3153932; doi:10.1371/journal.pone.0022350)
Supplement: Supporting Information S1 — (DOCX) [file pone.0022350.s001.docx]

**Supporting Information**

Details of Social Network Analyses

Two sets of analyses were conducted, one at the individual level and another at the group level to address how individual-level properties influence group-level dynamics. Below we describe these measures conceptually (also see Table 2). For operational definitions of network measures (e.g., groom in-degree, groom reciprocity, and reconciliation clustering), see Wasserman and Faust [1], Croft et al. [2] or as noted below. For the *individual level*, a number of measures were calculated to assess individuals’ influence in a network:

1. Individual Rank: Individual ranks were determined from behavioral management staff records of weekly observations of displacements and aggressive interactions and were supplemented by observations of agonistic interactions from this study. WinBugs software, a Bayesian approach [3], was used to analyze these data to generate a rank for each individual in each group.
2. Dominance Discrepancy: Individual rank does not measure how much *more* dominant an animal is to others in a group. To address this parameter, a measure of the extent to which individuals received more submission signals than expected was calculated by obtaining a z-score for each individual in each group where the number of submission signals (e.g., silent bared teeth display, turn away, move away, run away, scream) in *contexts of conflict* received by each individual was subtracted from the mean number of submission signals for the group and then divided by the standard deviation of the submission signals for the group. This z-scoring was conducted so that individuals could be compared across groups. Individuals with a high positive dominance discrepancy means greater separation from others in dominance at higher dominance levels and those with a high negative discrepancy means greater separation from others in dominance at lower dominance levels.
3. Social Power: a degree measure was calculated as a Shannon-like first-order entropic measure (for explanation of equation of first-order entropic measures, see references 4, 5). The proportion of the number of subordination signals or status signals (e.g., silent bared teeth display alone or with rump present, turn away or move away) a given individual received in *non-conflict contexts* (no overt threat was observed) was divided by the number of submission signals a given individual received in contexts of conflict for each individual in the social group. This proportion (p) was then used in a Shannon-like first-order entropic calculation as Σ p*log p which was summed for each individual and then multiplied by the number of subordination signals received, making it a weighted measure. This measure represents both the number of individuals from which an individual receives subordination signals (diversity) as well as the number of subordination signals received from those individuals (rate). This measure is similar to the ‘‘social power’’ measure in Flack’s work [5] but differs in that we chose to look at the *proportion of signals that were subordinate in relationship to those of submission*, as opposed to only the rate of subordination signals received. We think this distinction is important for comparison across rhesus macaques (as opposed to Flack’s pigtail macaques) that might receive subordination signals in non-conflict contexts due to their boldness in approaching/passing by other individuals (see Discussion). Adjusting this measure by the number of submission signals received gives us a more accurate estimate of how often individuals are *receiving unsolicited signals of subordination* or *status signals*. This measure represents how others in the network perceive individuals by measuring the extent to which individuals agree over the status of dominant individuals in a group or the perceived capability of individuals to use force [5,6]. The greater the consensus the more ‘‘powerful’’ an individual is. The values from each analysis obtained for each individual were then used to evaluate its association with other measures in the dataset.
4. Intervention Success: Conflict intervention is the involvement of a third-party in a conflict between others that is already occurring [7]. An intervention was defined by the involvement of a third party in an on-going conflict or within 5 seconds of the conflict’s initiation. A *successful intervention* is an intervention in which all conflict stops. Intervention success was calculated for each individual as the proportion of conflict interventions that were successful multiplied by the number of successful interventions for each individual ([# successful conflict interventions/total # interventions initiated by that animal]* # successful conflict interventions initiated by that animal). This measure takes into account how successful an individual is in stopping conflict but also the rate at which they do so (making it a weighted measure). Individuals with higher intervention success are those that intervene successfully with regularity.
5. Intervention Out-degree: an unweighted network measure of the diversity of individuals that an individual intervened upon [1].
6. Groom Betweenness Centrality: an unweighted network measure calculated in Netminer II [8] that represents the shortest paths that link every pair of individuals within a network and calculates the potential control that each individual has over flow within the network. This potential control is based on the probability that the focal individual lies on a path that links other pairs of individuals within the group while also taking into account alternative paths between pairs of individuals that pass through other non-focal individuals. Therefore, individuals with higher betweenness centrality are important in maintaining cohesiveness of the network through indirect ties [1].
7. Reconciliation In-degree: an unweighted network measure of the diversity of individuals from which an individual received reconciliation. Reconciliation is a repair mechanism involving affiliation such as grooming or huddling after conflict, which had to occur within 3 minutes of the termination of the original conflict.

For the *group level*, we calculated averages and standard deviations of the above measures as well as network measures aimed at assessing the connectedness of the group for different patterns of behavior: dominance interactions (fragmentation), status signaling (social power), aggression (intervention success) and affiliation (groom reciprocity and reconciliation clustering coefficient).

1. Hierarchy discrepancy: The number of submissions received by each individual in the group was calculated and then ranked and sorted by inverse rank; a scatter plot was generated and a natural log fit was conducted on the resulting plot generating a slope that represented the extent to which there was separation of the higher-ranking individuals from the lower ranking individuals, termed “hierarchy discrepancy”. The negative slope of that fit value was converted to a positive value, which was used in subsequent analyses. A higher value means a greater hierarchy discrepancy in the group.
2. Average Power: An averaged measure of social power as described above.
3. Average Intervention Success: An averaged measure of intervention success as described above.
4. Displacement Fragmentation: Fragmentation is an inverse measure of the amount of connectedness or connection redundancy in a network. In the context of displacements (the approach by one individual causes another individual to move/run away and is used to establish and maintain dominance relationships), greater redundancy in dominance interactions among individuals (thus representing lower fragmentation) should result in less ambiguity over dominance relationships. Less ambiguity over dominance relationships should result in lower levels of aggression during displacement activities, as individuals should concede more quickly when dominance relationships are known and stable. Fragmentation is defined as the proportion of mutually reachable nodes as each node is removed or unconnected from the network and calculated using the F measure as described in [9] and [10]. This measure was calculated in UCINET [10].
5. Groom Reciprocity: Reciprocity represents the extent to which individuals in a group reciprocate the connections or ties between them, where the connections between individuals are both incoming and outgoing. It is a useful measure of cohesion in primate groups (where grooming ties are quite dense), because the connections are mutual; there are more paths and directions through which information can flow in a network. As such, it is thought that groups with higher reciprocity are more “stable” than those with asymmetrical relationships. In our context, groom reciprocity represents the extent to which individuals in a group reciprocate the grooming of partners that had previously groomed them and was calculated in Netminer II.
6. Reconciliation Clustering Coefficient (Network Average): The network average clustering coefficient [1] is a network measure that evaluates the extent to which a network is composed of transitive relationships and thus measures the extent to which the network is clustered and closely connected. It also represents a measure of group cohesion as highly connected networks provide higher flow of information than less connected networks [2]. We calculated the reconciliation clustering coefficient in Netminer II to examine the extent to which groups exhibited these transitive connections in their reconciliation behavior which indicates higher cohesion and less separation among individuals in these networks.
7. Average Conflict Length: a measure of the average number of transactions or instances of conflict in a conflict event that occurred within 5 seconds of each other in a group. This measure represents the duration of conflicts, with longer conflicts leading to a potential increase in wounding.
8. Average Temperament: Averaged value of the factor scores of temperament for each personality type for each group across the study.
9. Average Contact Aggression: Averaged value of the amount of contact aggression (e.g. biting) within each group across the study.

Dependency of network measures across individuals

There is some concern in the literature over the use of network data in standard statistical models in that these types of relational data can violate the assumption of independence [11]. Therefore many investigators subject their dyadic matrices to permutation approaches to understand underlying relationships in the network structure. In contrast, our interest was not to use the dyadic data directly but as *summary network measures* at the individual level, such as social power or groom betweenness centrality, as a means for looking at associations between individual characteristics and the role individuals play within their social groups. We do not think that these summary network measures are overly dependent across individuals for the following reasons: (1) only one of our summary measures represents a dependent network measure, groom betweenness centrality; (2) although we use dyadic information to generate our measure of betweenness centrality, we do not expect that a high value for this network measure for some individuals necessarily means low values for other individuals (indeed in fully connected network, all individuals have a betweenness centrality of zero) and only at the positive extreme where a few individuals exhibit very high betweenness centrality (greatest value is 1), which is not represented in our data (range: 0.001-0.094); (3) the thresholding or filtering of weighted data to binary data as we conducted for betweenness centrality further reduces any potential dependency between individuals that does exist, which is not high enough to overthrow the results derived from assuming independence; and (4) any residual dependency across individuals in this network measure should be found mostly at the level of the matriline or cage, given macaque social structure, which is accounted for through the use of nested random effects in our analyses [12] (see below).

Comparisons of network measures across groups

Because our groups (networks) differed in size, we needed to account for the effect that a difference in the number of nodes alone might have on our group level network measures. Reciprocity, by definition, takes into account this difference as this measure represents a proportion of reciprocal ties to maximum possible reciprocal ties. For the measures of displacement fragmentation and reconciliation clustering coefficient where the number of nodes is an important issue, we conducted an expected value for each group generated from 1000 simulations of each measure. The method for calculating the expected measure was as follows: (1) we calculated the *density* (amount of connectedness; [1]) for the behavior in question (displacements, reconciliation) of each group; (2) we averaged the densities across groups to calculate a “normative” density for these groups; (3) we generated, for each group, 1000 Erdős–Rényi random graphs (in UCINET) using the averaged density and the original number of nodes from each group; and then (4) we subjected the random graphs generated for each group to the analysis of fragmentation (displacements) and clustering coefficient (reconciliation) to generate an averaged expected value for each measure for each group. The expected value (E) for each group was then subtracted from the observed value (O) and the final measure used in the analysis was the O-E measure in each case. We chose to develop this new method as opposed to other suggested methods [2] to generate expected values over concern that the number of edges in our networks existed because of the underlying differences in social dynamics across groups and not simply due to variation in the number of nodes in groups.

References

1. Wasserman S, Faust K (1995) Social Network Analysis: Methods and Applications. Cambridge University Press, Cambridge.
2. Croft DP, James R, Krause J (2008) Exploring animal social networks. Princeton University Press, Princeton, NJ.
3. Lunn DJ, Thomas A, Best N, Spiegelhalter D (2000) WinBUGS -- a Bayesian modelling framework: concepts, structure, and extensibility. Statistics and Computing 10: 325-337.
4. McCowan B, Hanser SF, Doyle, LR (1999) Quantitative tools for comparing animal communication systems: Information theory applied to bottlenose dolphin whistle repertoires. Animal Behaviour 57, 409–419.
5. Flack, J, de Waal F (2004) Dominance style, social power, and conflict. In: Kaumanns W (Ed.), Macaque Societies: A Model for the Study of Social Organization. Cambridge University Press, Cambridge, pp. 157–185.
6. McCowan B, Anderson K, Heagarty A, Cameron A (2008) Utility of social network analysis for primate behavioral management and well-being. Applied Animal Behaviour Science 109: 396-405.
7. Flack JC, Krakauer DC, de Waal FBM (2005) Robustness mechanisms in primate societies: a perturbation study. Proceedings of the Royal Society Biological Sciences Series B 272: 1091-1099.
8. Cyram (2005) Netminer II 2.6.0. Seoul: Cyram Co., Ltd.
9. Borgatti SP (2003) The key player problem. In: Breiger R, Pattison P (Eds.), Dynamic Social Network Modeling and Analysis: Workshop Summary and Papers. The National Academies Press, Washington, DC, pp. 241–252.
10. Borgatti SP, Everett MG, Freeman LC (2002) Ucinet for Windows: Software for Social Network Analysis. Harvard, MA: Analytic Technologies.
11. Wasserman S, Patterson, P (1996). Logit models and logistic regressions for social networks: I. An introduction to markov graphs and p*. Psychometrka 61, 401-425.
12. Stata (2009) Stata statistical software. Release 11.0 Reference H-P. Vol. 2. , Stata Corp, College Station, TX.
